# Caspase-1 as a regulatory molecule of lipid metabolism

**DOI:** 10.1186/s12944-020-01220-y

**Published:** 2020-03-06

**Authors:** Meseret Derbew Molla, Birhanu Ayelign, Gashaw Dessie, Zeleke Geto, Tesfahun Dessale Admasu

**Affiliations:** 1 Department of Biochemistry, School of Medicine, College of Medicine and Health Sciences, University of Gondar, Gondar, Ethiopia; 2 Department of Immunology and Molecular Biology, School of Biomedical and Laboratory Science, College of Medicine and Health Sciences, University of Gondar, Gondar, Ethiopia; 3 National Reference Laboratory for Clinical Chemistry, Ethiopian Public Health Institute, Addis Ababa, Ethiopia

**Keywords:** Caspase-1, Lipid metabolism, Inflammasome, Transcriptional factors

## Abstract

Caspase-1 is an evolutionarily conserved inflammatory mediated enzyme that cleaves and activates inflammatory cytokines. It can be activated through the assembly of inflammasome and its major effect is to activate the pro-inflammatory cytokines; interleukin 1β (IL-1β) and interluekine-18 (IL-18). In addition to IL-1β and IL-8, several lines of evidence showed that caspase-1 targets the substrates that are involved in different metabolic pathways, including lipid metabolism. Caspase-1 regulates lipid metabolism through cytokine dependent or cytokine independent regulation of genes that involved in lipid metabolism and its regulation. To date, there are several reports on the role of caspase-1 in lipid metabolism. Therefore, this review is aimed to summarize the role of caspase-1 in lipid metabolism and its regulation.

## Introduction

Caspases are a protein cleaving molecules grouped under the family of cysteine proteases that cleave their substrates following an aspartic acid (Asp) residue [1]. Their major role is to mediate programmed cell death since over expression of all catalytically active caspases can induce apoptosis [2]. It is also proved that caspase mediates the process of proliferation and inflammation [3]. Based on their function, caspases can be grouped into two major categories as apoptotic mediators (caspase-2, 3, 6, 7, 8, 9 and 10) and inflammatory mediators (caspase-1, 4 and 5) [4]. Inflammatory mediator caspases are a group of caspases that activate pro-inflammatory cytokines, which involved in the initiation of inflammation [5]. Inflammatory mediator caspases can also involve in cell death, particularly during metabolic disorders to overcome the stimulatory materials [6]. The most well-characterized inflammatory caspase is caspase-1, which is very important for the regulation of pro-inflammatory cytokines, such as IL-1β and IL-18 activation [7]. It was the first caspase reported as a protease in 1989 [8]. After 3 years in 1992, caspase-1 purified, cloned and sequenced, and found to be a new protein [9]. The caspase-1 expression is high in immune organs, such as spleen, lymph nodes and thymus due to their inflammatory mediated immune response following infection or damaged tissues [5]. Caspase-1 also expressed in adipose tissue, liver, and intestine because of their own immune privilege activity [10, 11]. These tissues are very important for energy metabolism [10]. Like other caspases, caspase-1 also presents as pro-caspase-1 or zymogen form in the tissue. Pro-caspase-1 gets activated by the proteolytic process through the assembly of cytosolic multi-protein complexes known as inflammasome [12].

Inflammasome assembly is an immediate multiprotein complex formation due to pathogen associated molecular patterns (PAMPs) or damage associated molecular patterns (DAMPs) detection through pattern recognition receptors (PRRs). This coordinates the host immune response against the danger sign through the activation of pro-inflammatory cytokines, such as IL-1β and IL-18 [12, 13]. Classical inflammasome complex contains three components; nucleotide-binding domain–like receptors (NLRs), absent in melanoma 2–like receptors (ALRs) or pyrin and the effector caspase (pro-caspase-1) [5]. Nucleotide-binding domain–like receptors are a cytosolic sensor, which detects microbial products or stress signals. Absent in melanoma 2–like receptors (ALRs), or pyrin, is an adaptor protein, which connects NLRs and the effectors. The NLR-associated N-terminal pyrin domain (PYD) interacts with the PYD of the apoptosis-associated speck-like protein containing a caspase recruitment domain (CARD) (ASC). Then the CARD domain of ASC interacted with the effector caspase (pro-caspase-1),which will be cleaved and activated itself and will further activate the target substrates to coordinate cellular activities [14, 15]. The most common and well-understood inflammasome is NLRP3 inflammasome also called NALP3 or cryopyrin, which activates by various PAMPs and DAMPs [16]. Some of these are uric acid crystals associated with Gout [17], extracellular adenosine triphosphate (ATP), calcium channel affecting marine toxin maitotoxin [18], ceramides [19], bacterial ribonucleic acid (RNA) [20], increased plasma free fatty acid [21, 22], high blood glucose level [23], and islet amyloid polypeptide [24]. Caspase-1 activation by these stimuli is the main intracellular danger sign; then, the target substrate will be activated and mediated the inflammatory process [25].

The main role of caspase-1 is activation of pro-inflammatory cytokine genes (pro IL-1β and pro IL-18) to express IL-1β and IL-18 protein, commonly called IL-1 converting enzyme because of its activity [8, 26]. On the other hand, it also brings inflammatory induced cell death or lytic form of programmed cell death called pyroptosis through proteolytic activation of Gasdermin D [27]. It is characterized by cellular lysis, the release of intracellular components, and inflammatory response, which is different from apoptosis and necrosis [15]. Caspase-1 can also associate with metabolic regulations, such as glucose homeostasis, body weight maintenance and lipid metabolism [28]. Caspase-1 regulates glucose metabolism by cleaving some of the glycolytic enzymes like aldolase, glyceraldehyde phosphate dehydrogenase, triose-phosphate isomerase, enolase and pyruvate kinase [29]. Similarly, it also regulates lipid metabolism through different mechanisms that can be cytokine-dependent or direct activation of regulatory transcriptional factors that involved in lipid metabolism [28]. However, the role of caspase-1in lipid metabolism has not yet been clearly addressed. Therefore, this review aimed to evaluate the non-canonical role of caspase-1, particularly on lipid metabolism.

### Role of caspase-1 in lipid metabolism

Affecting caspase-1 activity may lead to an imbalance in blood glucose homeostasis and lipid metabolism [30]. This is mainly because of two major reasons: caspase-1 regulates insulin sensitivity, and Il-1β and IL-18 regulates energy homeostasis. In support of this it has been shown that caspase-1 deficient mice develops obesity [3]. Lipid metabolism, following acute inflammation is to support innate immunity by activating lipid signaling involved in host immune system as well as redirecting lipoproteins to the sites of injuries [31]. However, chronic inflammation leads to severe changes in lipid metabolism, which leads to chronic disorders, such as atherosclerosis, obesity, type two diabetes mellitus, and other metabolic disorders [31, 32]. Caspase-1 activation through inflammasome is the main regulator of adipocyte function. Obesity induces inflammation and hence regulates caspase-1 activity. White adipose tissue (WAT) is the most relevant inflamed tissue caused by obesity-associated inflammation that promotes systemic inflammation and metabolic abnormalities due to suppression of insulin secretion and signaling [33]. On the other hand, caspase-1 deficient mice had reduced WAT mass with increased feces compared to the wild type mice, both consuming high fat diet [11]. This may suggest that caspase-1 can have a role in lipid absorption and de novo TG synthesis in the liver. Caspase-1 can also regulate lipid metabolism by a direct transcriptome effect with unclear mechanisms leading to the increment of serum TG level, limit action of adipocyte differentiation, and increment of insulin resistance [34]. Recent experimental findings also showed that IL-18 signaling (caspase-1 targeted substrate) lacked mice results impaired fat oxidation and ectopic lipid accumulation in skeletal muscles [35]. Thus, the effect of caspase-1 in lipid metabolism can be cytokine mediated or other multiple mechanisms.

**Fig. 1:**
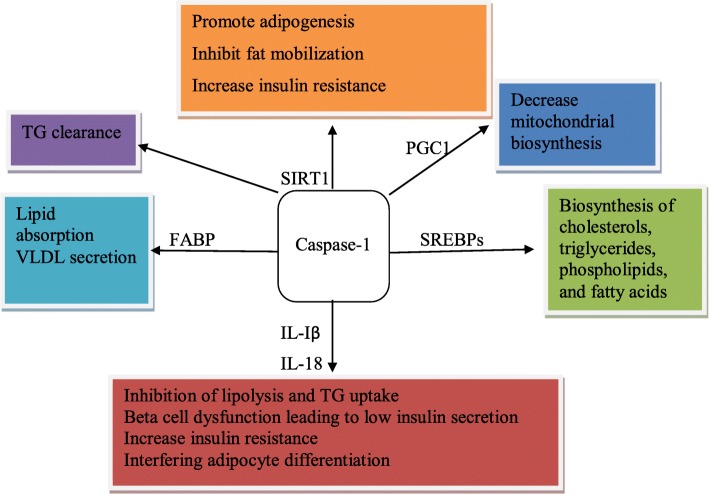
effect of caspase-1 in lipid metabolism.

Experimental investigations indicated that caspase-1 deficient mice had IL-1β or IL-18 signaling independent accelerated triglyceride (TG) clearance without alteration in lipid production and absorption, which will result in the decrease of steady-state circulating TG and fatty acid (FA) levels [28]. To the contrary, other research findings showed that caspase-1deficient mice had decreased TG absorption and hepatic very low density lipoprotein-triglyceride (VLDL-TG) production without reducing VLDL-apoprotein B (VLDL-apoB) production [11]. It had also reduced the uptake of TG derived FA in the liver, muscle and adipose tissue, with no effect on FA oxidation or FA uptake rather reducing intracellular FA transport, then leading to the shortage of lipid availability for the assembly and secretion of TG rich lipoproteins [11]. Therefore, caspase-1 has a different effect on lipid metabolism through various mechanisms (Fig. 1). From here, we take account that role of caspase-1 on lipid metabolism may be complicated with caspase-11 since Dixit VM and colleagues published that caspase-1 deficient mice may have defects in caspase-11. In addition, caspase-1 and caspase-11 are too close in the genome to be segregated by recombination, and caspase-11 coordinates both caspase-1 dependent and independent activities [13]. However, non-canonical stimulation of NLRP3 is critical for activation of caspase-1 but not caspase-11, which suggests that caspase-1 and caspase-11 can have distinct activators and target substrates. Therefore, we generalized that caspase-1 could have caspase-11 independent effect on lipid metabolism. And it will be further clarified in the upcoming studies.

*Caspase-1 can activate pro inflammatory cytokines and different transcriptional factors that involve in lipid metabolism. It activates pro IL-1β and IL-18, SREBPS, and FABP. And it inhibits the activation of SIRT1, PGC1α, and PGC1β to obstruct the target metabolic process. The activation of IL-1β and IL-18 promotes insulin resistance/beta cell dysfunction, decrease lipolysis/TG uptake and interferes adipocyte differentiation. The activation of SREBPS promotes the biosynthesis of cholesterols, TGs, phospholipids, and FAs. Activation of FABP manipulates lipid absorption and VLDL secretion. Active caspase-1 also decreases the biosynthesis of mitochondria via inactivation of PGC1α and PGC1β. Target substrates of SIRT1 also inactivates by active caspase-1 by inhibition of SIRT1. IL-1β, interleukin 1β: IL-18, interleukin 18: SREBPS, Sterol Regulatory Element-Binding Proteins: FABP, Fatty acid binding protein: SIRT1, sirtiun1: PGC1α, and PGC1β, peroxisome proliferator-activated receptor γ co-activator 1 family: TG, triglyceride: VLDL, very low density lipoprotein.

### Effect of caspase-1 in lipid absorption

The small intestine is the site of dietary fat absorption [36], and TG is the main dietary form of lipids [37]. The formation of mixed micelle from the end products of fat and bile salts can be absorbed at the brush border of enterocyte through passive diffusion or protein translocation mechanism [38]. Van Deepen et al. showed that caspase-1 deficient mice have increased feces with high FFA level with normal cholesterol and phospholipids and low WAT mass compared to the wild type mice, both consuming high fat diet. This suggests that caspase-1 deficiency can be related to lipid malabsorption or reduced intestinal FA absorption [11]. In their experiment, gene analysis of caspase-1 deficient mice showed that reduced fatty acid binding protein1 (FABP1) expression with no effect on the expression of CD 36, apoB (both hepatic and intestinal), and Microsomal triglyceride transfer protein (Mttp) [11]. This may result in reduced TG-rich lipoproteins secretion by decreasing intracellular FA transport without affecting the genes that involve in absorption as well as apoB secretion [11]. The fatty acid binding protein (FABP) is an important intracellular FA transporter protein used to carry intracellular hydrophobic FAs and monoacyleglycerol across the aqueous cytosol to the endoplasmic reticulum [39, 40]. The assembly of chylomicrons and lipidation of apoB takes place at endoplasmic reticulum and Golgi apparatus [36]. Fatty acid binding protein expression in the intestine can be liver type FABP/ FABP1 or intestinal type FABP/ FABP2. The latter is tissue specific whereas the former can also express in the liver and the kidney [36]. Microsomal triglyceride transfer protein is a very important protein for the assembly of chylomicrons and VLDL [40]. In contrast, other research finding showed that low plasma TG level in caspase-1 deficient mice was due to the increase of TG clearance, not the defect in intestinal FA absorption, chylomicron TG secretion, or intestinal absorption [28]. Here, we hypothesized that the contrary result may be due to confounding effects, experimental setup differences or the use of different composition of fat diet.

### Effect of caspase-1 in lipid transportation

Plasma TG level is determined by intestinal lipid absorption, hepatic VLDL secretion, and lipoprotein lipase (LPL) activity in the circulation. Both Van Diepen et al and Kotas et al tried to check whether caspase-1 had a role in the regulation of plasma TG level or not. Both of them found a low level of TG in the circulation of caspase-1 deficient mice compared to the wild type, but their justification was opposite [11, 28]. Van Diepen et al state that the defect in intestinal lipid absorption and hepatic VLDL secretion is the reason for the reduction of plasma TG in caspase-1 deficient mice. To the contrary, Kotas et al show the reduction of plasma TG is due to the accelerated clearance in the circulation, not the absorption or the secretion. The discrepancies between studies might be due to the reagents they used for the inhibition of LPL (Van Diepen et al use triton, and Kotas et al poloxamer).

Other studies also showed that pro-inflammatory mediated cytokine activation following infection results hypertriglyceridemia through the inhibition of LPL activity [41, 42]. Here, we suggested that caspase-1 activation can result hypertriglyceridemia. This is because of activation of IL-1β and IL-18, which are among pro-inflammatory mediated cytokine and can be activated by caspase-1. Other findings also showed that IL-1β activation/maturation affects insulin activity, which further results in decreasing the activity of LPL (stimulated by insulin) in the circulation, leading to increased plasma TG level and reduced plasma TG clearance [43]. Although the researcher did not clearly show caspase-1engegment, they stated that IL-1β activation (main target substrate of caspase-1), and we can suggest that there were effect of caspase-1 in this result. Caspase-1 interferes with insulin signaling on liver and adipose tissue and insulin production; hence, inflammasome activation of macrophage at the pancreatic β cell leads to IL-1β activation, which further results in β cell dysfunction and death [44, 45]. We can generalize that caspase-1 can have physiological role in the regulation of plasma TG clearance and peripheral tissue FA uptake through different mechanisms.

### Effect of caspase-1 in lipogenesis

Lipogenesis is the process of FA synthesis followed by TG synthesis by the esterification of glycerol with FA mainly in adipose tissue and liver during high carbohydrate diet consumption [46, 47]. Lipogenesis is stimulated by high blood glucose level and insulin for the purpose of fat storage in the form of TG [47]. High blood glucose level also activates caspase-1, which results IL-1β maturation in the adipose tissue and the liver [23]. Mature IL-1β interferes with insulin signaling and decreasing the expression of insulin signaling, leading to the decline of lipogenesis and fat accumulation [48]. In addition, IL-1β can also interfere with insulin production in the pancreatic β cells and can result in reduced lipogenesis [49]. Interlekuin-1β also inhibits the differentiation of adipocyte [50]; hence, adipogenesis or adipocyte differentiation improves by the inhibition of IL-1 signaling or caspase-1 activation.

Some other researchers have been reported that caspase-1 directly cleave the genes that involve in lipid metabolism, such as sterol regulatory element binding proteins (SREBPs) [51, 52], Peroxisome proliferator–activated receptor γ (PPARγ) [53], and the inactivation of PGC1α and PGC1β gene expression at adipose tissue, which are very important for mitochondrial biosynthesis and energy expenditure [54]. The expression of PGC1α and PGC1β gene are high in caspase-1 deficient mice [55]. Furthermore, active caspase-1 also cleaves and inactivates SIRT1 to indorse metabolic disorders [56, 57].

Sirtuin1 (SIRT1) is a nuclear NAD^+^ dependent protein deacetylase, which controls various transcriptional factors and co-factors involved in systemic metabolic homeostasis [58]. Degrading SIRT1 through caspase-1 activation results in the inactivation of the pathway downstream of SIRT1 [56]. In adipose tissue, SIRT1 inactivates the expression of PPARγ to inhibit adipogenesis, to promote fat mobilization [59], and to improve insulin sensitivity by deacetylating nuclear factor kappaB (NFκB) [60]. In the liver, SIRT1 also deacetylates and inactivates SREBP1 which leads to a decrease in FA and cholesterol biosynthesis during fasting [61, 62].

Sterol Regulatory Element-Binding Proteins are a subclass of basic helix–loop–helix–leucine zipper (bHLH-LZ) transcription factors that regulate gene expression involved in the biosynthesis of cholesterols, TGs, phospholipids, and FAs [63]. In mammals including humans, there are two SREBP genes (SREBP-1 and -2) that express three major SREBP proteins (SREBP-1a, SREBP-1c and SREBP-2) [64]. Different research findings showed that caspase-1 had a direct role on it. Caspase-1 activation of SREBPs promote cell survival through lipid metabolism following pore forming bacterial toxins by facilitating membrane repair [51]. Caspase-1 deficient mice have reduced expression of SREBP-1 both in the liver and intestine. Similarly, expression of hepatic SREBP-1c and intestine SREBP-2 and their downstream gene fatty acid synthase (fasn) is decreased in caspase-1 deficient mice [11]. fasn is an important regulator of fatty acid synthesis and lipogenesis [65]. Therefore, caspase-1 activation is one of the main regulators of lipogenic genes involved in TG as well as cholesterol metabolism.

### Effect of caspase-1 in lipolysis and β-oxidation

Lipolysis is the process of releasing FFA and glycerol from stored TG during starvation or prolonged exercise [66]. Insulin signaling is an important regulator of lipolysis. Caspase-1 activates pro IL-1 β and active IL-1 β inhibits insulin signaling pathways [49, 67]. Inflammatory cytokines, such as tumor necrotizing factor α (TNFα) and IL-1β induce lipolysis independent of catecholamine-induced lipolysis in adipose tissue [68, 69]. On the other hand, IL-18 (one of the substrate for caspase-1) has an opposite effect to IL-1β [49] and anti-obese effect to prevent metabolic dysfunction [70]. Interlekuin-18 deficient or IL-18 receptor knockout (Il18r1−/−) mice have obesity associated insulin resistance during high fat/protein diet [71, 72]. Maturation of IL-18 by NLRP1 inflammasome prevents obesity and metabolic syndrome by increasing insulin sensitivity and peripheral FA uptake [70], but most commonly IL-1β masks the activity of IL-18 during systemic inflammation.

Surprisingly, another study showed that the expression of genes involved in FA oxidation in both the wild type and caspase-1 deficient mice are the same [11]. The genes that checked were peroxisome proliferator activated receptor alpha (Ppara), PPAR-gamma coactivator1-beta (Ppargc1b), carnitine palmitoyltransferase 1 (Cpt1) and mitochondrial Carnitine/acylcarnitine translocase (Slc25A50) [11]. In line with this, in vivo intravenous injection of active IL-1 family to mice did not associate with the increment of serum FFA, which indicates that lipolysis is not stimulated by it [41]. The discrepancies between studies may be due to experimental setup variations, differences in the compositions of dietary fat (atherogenic diet) that they used, or differences in the microbiome of animals between facilities that could contribute to the phenotype discrepancy since gut microbiome homeostasis can be regulated by inflammasomes and IL-18. Although different researchers found different results, caspase-1 and the inflammasome directly or indirectly have a physiological role in energy and lipid metabolism.

## Conclusion

Caspase-1 is a well-known and well-studied inflammatory mediated caspase which cleaves and activates the inflammatory cytokines; proIL-1β and proIL-18. The activation of caspase-1 itself mediated through the assembly of multimeric protein complex called inflammasome, which stimulates by different PAMPs and DAMPs. Caspase-1 can involve in the metabolism of TG through the activation of cytokines, like IL-1β and IL-18. IL-1β affects TG metabolism by interfering insulin signaling and production. It can also directly inhibit LPL in the circulation. IL-18 has physiological significance by increasing insulin sensitivity and protecting metabolic dysfunction. Most researchers found opposite effect with IL-1β, but masked by IL-1β during inflammatory disorders. Caspase-1 deficient mice resulted in increased TG clearance from the circulation without the involvement of IL-1β and IL-18 metabolism. In contrast, other findings show the effect of caspase-1 in intestinal TG absorption and hepatic lipid secretion without a change in TG clearance. It can also activate or inactivate some genes that involve in TG metabolism, such as FASN, SREBP, PPARγ, PGC1α and PGC1β. In addition, it can cleave and inactivate SIRT1 to inhibit its targeted metabolic pathways.

## Abbreviations

DAMPs: Damaged associated molecular patterns
FA: Fatty acid
FABP: Fatty acid binding protein
FFA: Free fatty acid
IL: Interleukin
LPL: Lipoprotein lipase
PAMPs: Pathogen associated molecular patterns
SREBPs: Sterol regulatory element binding proteins
TG: Triglyceride
WAT: White adipose tissue
